# Clinical characteristics of children treated with high-frequency oscillation ventilation

**DOI:** 10.1097/MD.0000000000046288

**Published:** 2025-11-28

**Authors:** Lihua Wen, Xiaohuan Guo, Yiyu Yang, Run Dang, Chunmin Zhang, Feiyan Chen

**Affiliations:** a Pediatric Intensive Care Unit, Guangzhou Women and Children’s Medical Center, Guangzhou Medical University, Guangzhou, China; b Guangzhou Medical University, Guangzhou, China.

**Keywords:** CareFusion 3100A/B, clinical features, high-frequency oscillation ventilation, pediatric intensive care unit, respiratory support

## Abstract

High-frequency oscillation ventilation (HFOV) is a commonly used therapeutic modality in the pediatric intensive care unit, and this study aims to investigate the clinical characteristics of children treated with HFOV. The clinical data of children admitted to the PICU of Guangzhou Women and Children’s Medical Center from January 1, 2017 to December 31, 2022, who were treated with HFOV, were retrospectively collected and the clinical features data were analyzed. A total of 52 children treated with HFOV were included in this study, with a higher number of infants compared to older children (43 infants vs 9 older children, *P* <.05). The clinical manifestations of the children were multi-systemic diseases. 73.1% of the children were diagnosed with severe pneumonia, 23.1% had pulmonary hypertension, 25.0% were diagnosed with septicemia, and more than half of the children had underlying diseases in different systems, which required additional advanced support during treatment. In terms of pathogenesis, adenovirus (13.5%), Staphylococcus aureus and Mycoplasma pneumoniae (11.5%), and fungal infections (23.1%) were frequently identified, with many children suffering from mixed infections. Complications were notable; 19.2% of the children experienced air leakage, while 63.5% had mucus plugging. Finally, 46.2% of the children improved and were discharged, while 36.5% unfortunately did not survive. Surviving infants had a lower average peak airway pressure with HFOV than the fatal group (*P* <.05), Additionally, the oxygenation index values were lower, and the *P*/*F* values (PaO2/FiO2 ratio) were higher in the survivors than in the fatal group (*P *<.05). Children treated with HFOV were predominantly infants and young children under 36 months old. These patients exhibited high mortality rates and impacted on multiple systems throughout the body, often with mixed infections. These children required more significant medical support and longer treatment times.

## 1. Introduction

In the pediatric intensive care unit (PICU), high-frequency oscillation ventilation (HFOV) is a commonly used ventilation therapy. It achieves lung protection mainly through tidal volumes below the normal lower limit but at a higher-than-normal respiratory rate. By employing a high-frequency, low tidal volume approach to gas delivery, HFOV minimizes lung tissue damage in children, decreases alveolar pressure, and ensures constant airway pressure throughout treatment. This significantly reduces the risk of barotrauma and volutrauma.^[1,2]^ Currently, HFOV is predominantly applied in neonatology for conditions such as neonatal respiratory distress syndrome and persistent pulmonary hypertension of the newborn (PPHN).^[3,4]^

As a widely used treatment in the PICU, HFOV serves primarily as a rescue intervention for children suffering from acute respiratory distress syndrome (ARDS) or severe pneumonia when conventional ventilator therapy fails. It can be applied to pediatric patients with cardiovascular diseases and congenital diaphragmatic hernia.^[5]^ The Oscillation (OSCAR) trial targeting ARDS from 2007 to 2012 found no difference in 30-day mortality.^[6]^ In contrast, the OSCILLate trial, which examined the early administration of HFOV for ARDS during the same timeframe, was prematurely terminated due to observed higher in-hospital and 60-day mortality rates among the HFOV group.^[7]^ Furthermore, a meta-analysis conducted in 2020 revealed that HFOV did not improve outcomes, including mortality rates, compared to conventional mechanical ventilation.^[8]^ Currently, there is a lack of sufficient pediatric data to guide the timing and outcomes associated with HFOV utilization in the PICU, leading to considerable variability in clinical practice.^[9,10]^ This inconsistency makes it difficult for clinicians to determine the optimal timing and potentially resulting in missed therapeutic opportunities. By enhancing the application of HFOV within the PICU, there is a significant opportunity to improve outcomes for children, reduce hospital mortality rates, and minimize complications.

Therefore, this study aims to retrospectively analyze the clinical data of 52 critically ill children who received HFOV in the PICU at Guangzhou Medical University Affiliated Women and Children’s Healthcare Center from 2017 to 2022. The focus will be on examining their clinical characteristics, the diagnosis and treatment processes, and any complications encountered, ultimately providing valuable insights into the management of critically ill pediatric patients requiring HFOV.

## 2. Methods

### 2.1. Ethics statement

This study adhered to the ethical standards set by the involved institutions, with approval granted by the Ethics Committee of Guangzhou Women and Children’s Medical Center (Approval Number: 198A01/2024). The official ethics approval documents were provided as Supplementary Materials, Supplemental Digital Content, https://links.lww.com/MD/Q811. All procedures involving human participants were conducted following the ethics standards of these committees and in alignment with the 1964 Helsinki Declaration and its later amendments or equivalent ethical standards.

### 2.2. Study population

**Figure 1.**
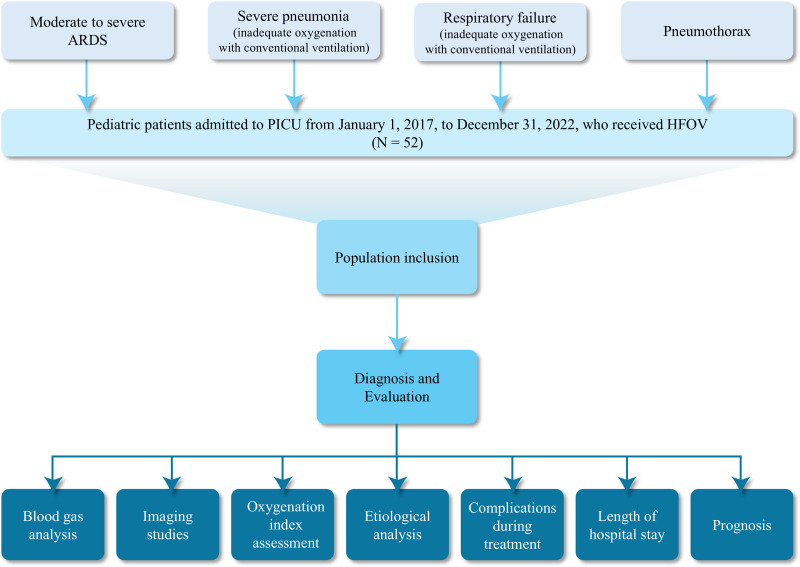
Flow chart of the study population. ARDS = acute respiratory distress syndrome, HFOV = high-frequency oscillatory ventilation.

A total of 52 pediatric patients who were admitted to the PICU of Guangzhou Medical University Affiliated Women and Children’s Medical Center between January 1, 2017 and December 31, 2022, and received HFOV (CareFusion 3100A/B) were included in this study. The data and information of the pediatric patients are all sourced from the electronic medical record system, and the data included in the analyzed pediatric medical records are complete. The indications for HFOV in our hospital were as follows: moderate to severe ARDS; severe pneumonia patients who could not maintain adequate oxygenation with conventional mechanical ventilation; respiratory failure due to various causes where conventional mechanical ventilation could not maintain adequate oxygenation; patients with pneumothorax, as detailed in Figure 1. Among the enrolled children, the final outcome is defined as the survival group upon discharge, and the death group upon in-hospital occurrence.

### 2.3. Date collection

The general clinical data collected from the patients included general information (gender, age, total length of hospital stay, PICU length of stay, duration of HFOV, complications, prognosis, and outcome), ventilator parameters, laboratory test indicators, and pathogen test data.

### 2.4. Statistical analysis

Data analysis was performed using SPSS 23.0 software (Chicago). Categorical data were described using rates, frequencies, relative ratios, and modes. The chi-square test was implemented for inter-group difference analysis. *P* <.05 indicated a statistically significant difference. If the measurement data were normally distributed and passed the homogeneity of variance test, they were expressed as mean ± standard deviation (*x *± *s*), and parametric tests were used for statistical analysis. If the measurement data were not normally distributed, they were described using median and interquartile range M (Q1, Q3), and nonparametric tests were used for inter-group comparison.

## 3. Results

### 3.1. Baseline characteristics

A total of 52 infants who received HFOV were included in this study. The median age of the infants was 9 months, with an interquartile range of 26.75 months (3 to 29.75 months). The most common age among infants was 4 months. Among these infants, 29 were male (55.77%) and 23 were female (44.23%). The study comprised 43 infants aged 1 to 36 months, while 9 older children were above 36 months of age. The number of infants was significantly greater than that of older children (*t* = 10.752, *P* <.05).

### 3.2. Clinical manifestations

#### 3.2.1. Respiratory system

In this study, 73.1% (38 out of 52) of patients were diagnosed with severe pneumonia, among which 17.3% (9 out of 52) were classified as respiratory distress syndrome and 76.9% (40 out of 52) developed into respiratory failure. Bronchopulmonary dysplasia was present in 25.0% (13 out of 52) of patients, and pulmonary hemorrhage in 3.8% (2 out of 52).

#### 3.2.2. Cardiovascular system

In this study, 23.1% (12 out of 52) of patients were diagnosed with pulmonary hypertension, and 38.5% (20 out of 52) had concomitant congenital heart disease, including ventricular septal defect in 19 cases (36.5%), valvular disease in 7 cases (13.5%), patent ductus arteriosus in 6 cases (11.5%), pulmonary venous anomalies in 2 cases (3.8%), and complete transposition of the great arteries in 2 cases (3.8%).

#### 3.2.3. Other systems

**Figure 2.**
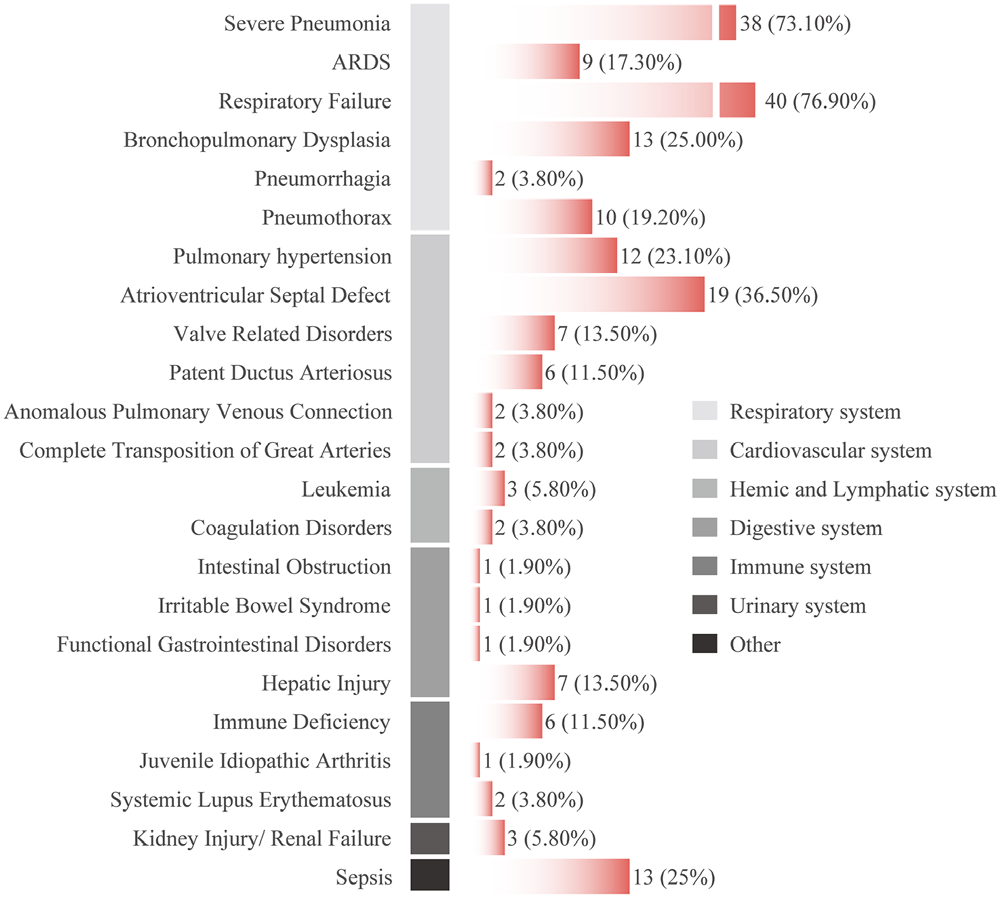
Clinical manifestations of various systems in pediatric patients.

In this study, 25.0% (13 out of 52) of patients were diagnosed with sepsis, while 9.6% (5 out of 52) had hematological diseases including leukemia, chemotherapy-induced bone marrow suppression, and coagulation disorders. Additionally, 7.7% (4 out of 52) of patients had gastrointestinal diseases such as intestinal obstruction, gastrointestinal dysfunction, and allergic enteritis. 15.4% (8 out of 52) of patients had immune system diseases, mainly immunodeficiency, but also including juvenile idiopathic arthritis and systemic lupus erythematosus. Furthermore, 13.5% (7 out of 52) of patients had varying degrees of liver function impairment, and 5.8% (3 out of 52) had varying degrees of kidney function impairment. Specific details can be found in Figure 2.

### 3.3. Pathogen testing

**Figure 3.**
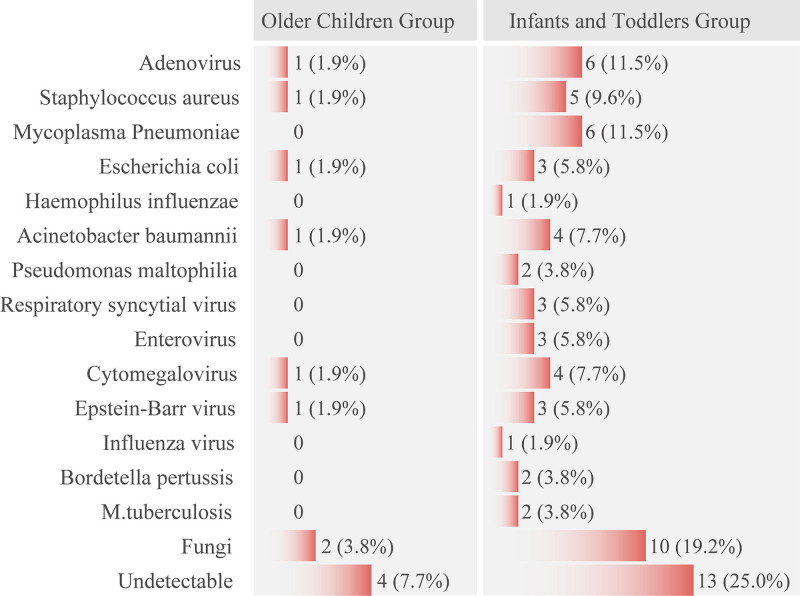
Infection etiology in pediatric patients (number of cases [%]).

Among the included patients, the main pathogens detected were as follows: adenovirus in 7 cases (13.5%), Staphylococcus aureus and Mycoplasma pneumoniae in 6 cases each (11.5%), respiratory syncytial virus and enterovirus in 3 cases each (5.8%), Escherichia coli in 4 cases (7.7%), Acinetobacter baumannii in 5 cases (9.6%), cytomegalovirus in 5 cases (9.6%), Epstein-Barr virus in 4 cases (7.7%), and fungal infections (including Pneumocystis jiroveci, Aspergillus, and Candida) in 12 cases (23.1%). In 17 cases (32.7%), no pathogens were identified, as detailed in Figure 3. Among all patients with HFOV, mixed infections were the most common, with 34 cases (65.4%), indicating that the condition of children using HFOV is more complex.

### 3.4. Treatment process

Among the 52 infants who received HFOV, the plateau pressure (PF) before HFOV was (81.05 ± 63.26). Nasotracheal intubation was used in 36 cases (49.2%), bronchoscopy in 15 cases (28.8%), and hemopurification in 11 cases (21.2%), with a single session of hemopurification being the most common. Additionally, 9 cases (17.3%) required extracorporeal membrane oxygenation (ECMO) due to unstable vital signs, and the mortality rate in the ECMO group was 44.4%. The duration of HFOV ranged from 1 to 445 hours, with an interquartile range of 51.89 hours (43.88–95.77 hours). Patients were divided into survival and death groups. There were no statistically significant differences between the 2 groups in terms of gender, age, weight, and underlying diseases. However, the APACHE II score at admission was significantly higher in the death group compared to the survival group (*P* <.05), as shown in Table 1.

**Table 1:** Comparison of clinical features between surviving and deceased HFOV pediatric patients.

	Survival group (n = 33)	Mortality group (n = 19)	χ^2^/Z	*P*
Male (n/%)\	17 (51.5%)	12 (63.2%)	4.76	.19
Age (M [P25, P75])	24.5 (4, 28.5)	34 (2, 36)	−0.905	.365
Weight (M [P25, P75])	6.14 (4.51, 10.65)	9.7 (3.9, 13.6)	−0.076	.939
APACHE II score (x ± s)	13.27 ± 3.46	16.63 ± 2.83	−3.59	.001
Underlying conditions (n/%)	21 (63.6%)	8 (42.1%)	7.23	.065

HFOV = high-frequency oscillation ventilation.

### 3.5. Comparison of treatment outcomes

Comparing the 2 groups, we found that the highest mean airway pressure during HFOV was significantly lower in the survival group compared to the death group (*P* <.05). The highest oxygenation index (OI) during monitoring was also significantly different between the survival and death groups (*P* <.05), with the survival group showing higher values. Additionally, the lowest *P*/*F* ratio was significantly higher in the survival group compared to the death group (*P* <.05), as shown in Table 2. Among the survival group, only 4 patients did not transition from high-frequency ventilation to conventional ventilation. Three of these 4 patients had congenital heart disease, while the remaining one was discharged at their own request. The airway pressure set when weaning from high-frequency ventilation was (20.87 ± 4.42), the monitored OI was (7.08 ± 2.84), and the monitored *P*/*F* ratio was (249.81 ± 110.17). After weaning from the ventilator, 17 patients required noninvasive ventilation (32.7%), and 23 patients were successfully weaned from high-frequency ventilation (44.2%).

**Table 2:** Comparison of outcomes between 2 groups during HFOV treatment.

	Survival group (n = 33)	Mortality group (n = 19)	*t*	*P*
Peak mean airway pressure	24.46 ± 4.98	30.99 ± 4.96	2.98	.005
Highest O/I	22.77 ± 11.61	38.42 ± 12.58	4.20	0
Lowest *P*/*F*	63.85 ± 31.93	41.09 ± 21.86	2.49	.016

HFOV = high-frequency oscillation ventilation.

### 3.6. Complications during treatment

Among the 52 infants receiving high-frequency ventilation, 10 (19.2%) developed pneumothorax. Seventeen patients (32.7%) experienced cardiopulmonary resuscitation during high-frequency ventilation. Thirty-three patients (63.5%) developed mucus plugging during treatment. No cases of intracranial hemorrhage were observed as a complication of HFOV. This is consistent with the common complications of HFOV, and in future clinical work, active treatment of the primary disease should be carried out, as well as avoiding thick sputum and regularly reviewing chest X-rays.

### 3.7. Length of stay and prognosis

The 52 infants in the study had a length of stay in the PICU ranging from 1 to 305 days, with an interquartile range of 37 (19–55) days. Twenty-four patients (46.2%) were discharged after recovery, while 19 died (36.5%), which included 16 infants and 3 older children. And 9 patients (17.3%) were discharged at their own request.

## 4. Discussion

HFOV is a commonly used treatment modality in the PICU. It generates oscillating airflow in the airway through a vibrating membrane or piston, delivering high oxygen and humidified airflow to the patient’s alveoli with low tidal volume, effectively re-expanding collapsed alveoli and improving lung oxygenation, allowing for the delivery or removal of much smaller tidal volume than anatomical dead spaces, and preventing carbon dioxide retention.^[11,12]^ Meanwhile, HFOV forms sustained low pressure within the alveoli, which significantly reduces the risk of barotrauma and volumetric injury.^[13,14]^

HFOV is a standard treatment for PPHN.^[13]^ The age range of patients receiving HFOV in this study spanned from 1 month to 15 years. However, when grouped based on age at admission, with 36 months as the cutoff, the infant group was significantly larger than the older child group. This suggests that the design and operating principles of high-frequency ventilators are better suited to meet the physiological needs of infants. Specifically, the ability to provide small tidal volumes and lower continuous positive airway pressure promotes oxygenation, improves gas exchange, and protects the fragile lung tissue of infants, who typically have lower body weight and smaller lung volumes. These factors make HFOV particularly valuable for infants.^[15]^ Owing to these characteristics, our study found that surviving patients on HFOV had lower peak airway pressures compared to those who did not survive, and the lowest measured *P*/*F* ratios were higher in the survival group. However, there was no statistically significant difference in the OI between the 2 groups. This may be attributed to the relatively small sample size and the fact that some patients with high OI values eventually required ECMO due to deteriorating vital signs or other complications.

The 2023 pediatric ARDS (pARDS) guidelines recommend that HFOV should be particularly considered for moderate to severe cases of pARDS.^[16]^ In our study, 73.1% of pediatric patients were diagnosed with severe pneumonia. Among them, 17.3% met the criteria for respiratory distress syndrome, and 76.9% progressed to respiratory failure. Additionally, 23.1% were diagnosed with pulmonary hypertension, 38.5% had congenital heart disease, and 25.0% experienced sepsis. These results indicate that the use of HFOV in the PICU should not be limited to neonatal respiratory conditions like respiratory distress syndrome and pulmonary hypertension alone. Instead, HFOV can be a crucial therapeutic option for patients experiencing respiratory failure due to a variety of single-system or multi-system diseases, including congenital heart defects. This highlights the need for a broader approach in considering HFOV for critically ill pediatric patients.

Almost all patients who underwent HFOV therapy subsequently required conventional mechanical ventilation before being weaned from mechanical support. Only 4 patients in this study were directly weaned from HFOV, of whom 3 had underlying congenital heart disease. These patients had relatively stable lung conditions and were able to be weaned from HFOV once their primary condition was addressed. The remaining patient was discharged against medical advice. As demonstrated by the results of this study, patients receiving HFOV in the PICU often have multi-system diseases rather than isolated conditions. Consequently, these patients may also require bronchoscopy and hemodialysis. Despite aggressive treatment, 9 patients in this study ultimately required ECMO to maintain adequate oxygenation.

A significant proportion of patients included in this retrospective study were diagnosed with severe pneumonia and sepsis. The etiological spectrum of these infections encompassed bacteria, fungi, and viruses, with adenovirus, Staphylococcus aureus, Mycoplasma pneumoniae, and Pneumocystis jirovecii being the most prevalent pathogens. These pathogens are known to cause severe pneumonia with high mortality rates, particularly in immunocompromised children. They can lead to multi-system organ failure and, if left untreated, can progress to sepsis or severe respiratory failure, potentially resulting in long-term sequelae.^[8,17]^ Our findings align with these established observations. Notably, 34 patients in our cohort exhibited mixed infections, predominantly involving 2 pathogens. This finding highlights the complex and severe nature of the underlying conditions in patients requiring HFOV for severe pneumonia or systemic infections. Previous large-scale retrospective studies have consistently demonstrated that mixed infections are a significant determinant of disease severity in pediatric pneumonia, with various combinations and patterns.^[18,19]^

Current research suggests that the incidence of barotrauma associated with high-frequency oscillatory ventilation (HFOV) is not significantly different from that of conventional mechanical ventilation.^[20]^ In our study, 10 cases of pneumothorax occurred, with 2 cases preexisting the initiation of HFOV. Previous retrospective studies have demonstrated that HFOV can effectively improve oxygenation, shorten the duration of mechanical ventilation, and accelerate pneumothorax absorption in patients with ARDS and pneumothorax, without increasing the incidence of adverse events.^[21]^ Therefore, it is crucial to continuously adjust airway pressures based on the patient’s condition to prevent complications arising from inappropriate ventilator settings. On the other hand, incomplete humidification, infection, and inadequate circulatory support during treatment can lead to inflammatory changes in the airway mucosa or mucus plugging, further inducing or exacerbating tracheobronchitis.^[22]^ In this study, 63.5% of patients experienced mucus plugging during HFOV therapy. This was determined by poor chest wall and abdominal vibrations, which improved after adequate airway care. We speculate that this was related to excessive sedation and neuromuscular blockade during treatment. Therefore, it is important for patients receiving HFOV to undergo daily sedation scoring and an assessment of the need for neuromuscular blockade. If neuromuscular blockade is required, it should be used judiciously, and airway care should preserve the patient’s cough reflex while maintaining ventilator synchrony. Additionally, adequate suctioning should be performed. It is also important to recognize that excessive airway pressure during HFOV can impair venous return, leading to hypotension.^[23]^ Furthermore, HFOV may influence intracranial venous return, resulting in fluctuations in intracranial pressure that could potentially cause intracranial hemorrhage. However, no cases of intracranial hemorrhage were observed during HFOV in this study. The 2 cases of intracranial hemorrhage were either present at admission or occurred during ECMO treatment. Other potential complications associated with HFOV include oxygen toxicity and ventilator-associated pneumonia.^[24]^

This study has certain limitations due to the restricted sample size. The insufficient number of samples may have prevented the effective detection of subtle yet real differences. Additionally, this smaller sample may lead to challenges when generalizing our findings to a wider population. In future research, we will continue to collect more data and increase the sample size to enhance the reliability of the study and the generalizability of its conclusions.

## 5. Conclusion

In summary, the significance of HFOV in the treatment of critically ill children is indisputable. Compared with conventional mechanical ventilation, HFOV effectively reduces airway pressure, significantly improves oxygenation, and decreases the incidence of barotrauma and volutrauma. However, patients requiring HFOV tend to be more critically ill, often infants, and involve multiple organ systems with mixed infections, indicating a higher complexity of care for these patients. Consequently, future research should focus on intensive management and care for HFOV patients, including larger sample size studies to explore their clinical characteristics, leading to a better understanding of HFOV, improved cure rates, reduced mortality and complications, and alleviated the economic strain on families and healthcare systems.

## Author contributions

**Conceptualization:** Lihua Wen, Feiyan Chen.

**Data curation:** Lihua Wen, Xiaohuan Guo.

**Formal analysis:** Lihua Wen, Yiyu Yang.

**Funding acquisition:** Lihua Wen, Run Dang, Feiyan Chen.

**Investigation:** Lihua Wen.

**Methodology:** Lihua Wen.

**Project administration:** Lihua Wen.

**Resources:** Lihua Wen.

**Supervision:** Lihua Wen.

**Validation:** Lihua Wen.

**Visualization:** Lihua Wen, Chunmin Zhang.

**Writing – original draft:** Lihua Wen, Xiaohuan Guo, Feiyan Chen.

**Writing – review & editing:** Lihua Wen, Xiaohuan Guo, Feiyan Chen.

## Supplementary Material


