# Supplementary material for: Clinical characteristics of children treated with high-frequency oscillation ventilation
Source: Medicine (Baltimore). 2025 Nov 28;104(48):e46288. doi: 10.1097/MD.0000000000046288 (PMC12662557; doi:10.1097/MD.0000000000046288)
Supplement: Supplementary file 1 [file medi-104-e46288-s001.pdf]

Guangzhou Women and Children's Medical Center Research Ethics Committee

Ethical Review Approval

Ethical Number: [2024] No. 198A01

**Project Title**	Clinical Characteristics of Children Treated with High-frequency Oscillation Ventilation		
**Principal Investigator**	Lihua Wen	**Department**	PICU
**Ethics Contact**	Lihua Wen	**Contact**	+86 18826444223
**Research Period**	2017.1.1-2027.12.31	**Review Category**	Expedited Review
**Review Documents**	* Application form for ethical review of biomedical research involving human subjects<br>* Researcher's CV<br>* Research protocol<br>* Informed consent form		
**Review Result**	According to the "Ethical Review Procedures for Biomedical Research Involving Human Subjects" (Order No. 11) of the National Health and Family Planning Commission of the People's Republic of China, the Declaration of Helsinki, and the International Ethical Guidelines for Biomedical Research Involving Human Subjects issued by the Council for International Organizations of Medical Sciences, the Ethics **Committee has decided to: Approve.**		
**Principal Reviewers**	Wei Jia , Yan Lin		
**Comments**	During the study, the investigator shall apply for follow-up review according to the frequency specified by the Ethics Committee. The applicant shall submit a research progress report one month before the deadline. The principal investigator must strictly use the approved informed consent form and research protocol. If the ethical review approval is invalidated and all clinical research (including statistical analysis) cannot be completed, please submit a continued review application one month before the expiration of this approval. If the study is completed within the validity period of the review, please submit a final research report. Any serious adverse events (SAEs) or unexpected adverse events involving		

扫描全能王
3亿人都在用的扫描App

	subjects or others that occur during the study should be reported to the Ethics Committee immediately. Any modifications to the research protocol or informed consent form, including changes to the research personnel, must be submitted for amendment review. If the study is prematurely terminated or suspended, please submit a suspension/ termination report in a timely manner. Any violation of the protocol or any situation that may adversely affect the rights and interests of the subjects, their health, or the scientific nature of the study, and any violation of the principles of scientific research ethics shall be reported in a timely manner.		
**Approval Validity Period**	2017.1.1-2027.12.31	**Follow-up Review Frequency**	12 months
**Signature of the Chairman (Vice Chairman) of the Committee**	Sitang Gong	**Date**	2024/Jul/05
**Ethics Committee**	Guangzhou Women and Children's Medical Center Research Ethics Committee		

Guangzhou Women and Children's Medical Center Research Ethics Committee
Contact: +86 020-38367270

Translation confirmed error-free

Lihua Wen, Xiaohuan Luo, Yiyu Yang, Run Dang, Chunmin Zhang, Feiyan Chen

扫描全能王
3亿人都在用的扫描App

广州市妇女儿童医疗中心科研伦理委员会

伦理审查批件

穗妇儿 科伦 批字[2024]第 198A01 号

**项目名称**	儿童重症监护室高频振荡通气患儿临床特点分析		
**负责人**	文俪桦	**专业部门/科室**	PICU
**伦理联系人**	文俪桦	**联系方式**	18826444223
**研究期限**	2017.1.1-2027.12.31	**审查类别**	**快速审查**
**审查文件**	1.涉及人的生物医学实验伦理审查申请表<br>2.研究者履历<br>3.研究方案<br>4.知情同意书		
**审查结果**	根据中华人民共和国国家卫计委第 11 号令《涉及人的生物医学研究伦理审查办法》以及《赫尔辛基宣言》和国际医学科学组织委员会颁布的《人体生物医学研究国际道德指南》等伦理原则，本伦理委员会审查决定为：**同意。**		
**主审委员**	贾炜、林艳		
**意见说明**	在研究进行过程中研究者请按照伦理委员会规定的跟踪审查频率，申请人在截止日期前 1 个月提交研究进展报告。研究负责人必须严格使用经审查同意的知情同意书文本和研究方案。如伦理审查批件失效时不能完成所有的临床研究（包括统计分析），请在本批件失效前一个月，递交持续审查申请。如研究结束并在审查有效期内，请递交研究结题报告。研究中发生涉及受试者或其他人风险的任何 SAE 或非预期的不良事件，应立刻报告本伦理委员会；任何研究方案、知情同意书的修改包括研究人员的变更，必须递交修正案审查申请；若提前终止或暂停研究，请及时提交暂停/终止研究报告。出现违背方案或可能对受试者权益/健康及研究的科学性造成不良影响等违背科研伦理原则的情况，应及时提交违背方案报告。		
**批件有效期**	2017.1.1-2027.12.31	**跟踪审查频率**	12 个月
**主任（副主任）委员签名**		**日期**	2024 年 7 月 5 日
**伦理委员会**	广州市妇女儿童医疗中心科研伦理委员会（盖章）		

广州市妇女儿童医疗中心科研伦理委员会

联系电话：020-38367270

扫描全能王
3亿人都在用的扫描App
